# Thirteen new records of ferns from Brazil

**DOI:** 10.3897/BDJ.3.e4421

**Published:** 2015-02-25

**Authors:** Thais Elias Almeida, Alexandre Salino

**Affiliations:** ‡ Universidade Federal do Oeste do Pará, Santarém, Brazil; § Universidade Federal de Minas Gerais, Belo Horizonte, Brazil

**Keywords:** Pteridophyta, floristic, disjunction, Amazon region, Andes

## Abstract

Thirteen fern species are reported for the first time for Brazil. Among the new records, eight are from Acre state (*Cyathea subincisa*, *Cyclodium trianae*, *Elaphoglossum stenophyllum*, *Hypoderris brauniana*, *Pleopeltis stolzei*, *Thelypteris arcana*, *Thelypteris comosa*, *Thelypteris valdepilosa*), two are from Pará state (*Polypodium flagellare*, *Tectaria heracleifolia*), one from Minas Gerais state (*Alsophila salvinii*), one from Ceará state (*Campyloneurum costatum*) and one from Bahia state (*Thelypteris rolandii*). Part of the species shows a disjunct occurrence or illustrates floristic relations between Brazilian and Andean Mountains or Central American Mountains.

## Introduction

Brazil figures as one of the most diverse countries in the world and harbors distinctive ecosystems such as Atlantic Forest, Cerrado and Amazonia (Veloso et al. 1991). The indigenous flora has been studied since the 18^th^ century, with thousands of species described and documented through the years. Notwithstanding, the country is still far from consolidating its botanical knowledge (Sobral and Stehmann 2009) and collection efforts are still necessary to generate data on species distribution, and provide basis for studies on centers of endemism and richness, patterns of geographic distribution and accurate information on species threat level (Sobral and Stehmann 2009).

As a result of working effort of several taxonomists, exactly 150 years after the publication of Flora Brasiliensis’ first volume, Forzza et al. (2010) published a list of Brazilians’ known plant species, an attempt that represented an initial step in gathering information and trying to answer the question of how diverse Brazilian flora is. This checklist is also available online (http://reflora.jbrj.gov.br/jabot/listaBrasil/ConsultaPublicaUC/ConsultaPublicaUC.do) and is periodically updated to include taxa and taxonomic novelties.

Although this publication represented a remarkable starting point, it also depicted a wide range of problems, such as lacking of studies (Salino and Almeida 2008a​) and sampling for several taxonomic groups, as well as those regarding taxonomic issues (Salino and Almeida 2008a, Prado and Sylvestre 2010). Additionally, the list highlighted the lack of knowledge and the insufficient and often biased sampling efforts performed in Brazil (Forzza et al. 2010, Prado and Sylvestre 2010).

As an example of the lack of an adequate sampling, the works of Nelson et al. (1990) and Hopkins (2007) showed how biased and fragmentary are collections in brazilian Amazon. The results presented by Hopkins (2007) indicate large areas of missing information where uncollected and even undescribed species probably lies, and are therefore the ones where additional collection efforts will mostly likely bring up novelties. Although occurrence of new records for brazilian Amazon are normally expected due to the lack of studies performed in this portion of the country (Forzza et al. 2010), finding new records in Southeastern Brazil is somewhat surprising as it holds some of the largest and most traditional plant research centers in the country (Sobral and Stehmann 2009). In this context, it is clear that even easily accessible areas close to major research centers may be still far from an ideal sampling, making it harder to precisely define geographic distribution of many taxa.

The few states that have so far published lists and pteridophyte floras are Acre (Prado and Moran 2009), Santa Catarina (Gasper et al. 2012) and São Paulo states (Prado and Hirai 2011). Despite of these recent sampling efforts, new records and species have been constantly discovered in these and other states, as a result of increasing collection efforts in unexplored or poorly surveyed areas (Lopes et al. 2003, Pietrobom and Barros 2003, Pietrobom et al. 2004, Barros et al. 2005, Pereira et al. 2005, Costa et al. 2006, Labiak and Prado 2007, Biral and Lombardi 2012, Biral and Prado 2012, Carvalho et al. 2012, Góes-Neto and Pietrobom 2012, Dittrich and Souza 2013).

Ferns present a peculiar geographic distribution, as they have light-weighted wind-dispersed spores that can easily cross barriers (Tryon 1986, Moran 2008). These plants normally present wide ocurrence ranges and disjunct populations (Tryon 1972, Page 1979a, Kessler 2010) - species can have populations separated by more than 500 miles (Tryon 1972); environmental conditions seems to be intimately associated with habitat circumscription to most ferns and lycophytes species (Page 1979a). Although they are known to occur in a wide variety of habitats, tropical regions hold higher species richness than temperate ones: Southeastern Asia and Tropical America, for example, account for ca. 60% of fern species (Moran 2008). This richness, however, is unevenly distributed: middle-elevation mountains (800-2000 m) hold the richest ferns communities and the largest number of endemic species (Page 1979b, Moran 2008). As an example, primary centers for Neotropical ferns, as defined by Tryon (1972) (areas with higher species number and higher endemism), correspond to the main mountain ranges in Tropical America: Mexico, Andes, and Eastern Brazil. This geographic distribution unevenness is usually explained by the greater environmental heterogeneity present in mountainous regions (Moran 2008, Kessler 2010).

The aim of this paper is to present 13 species previously unknown to occur in Brazil.

## Materials and methods

Taxonomic identifications were based on specific literature or comparisons with material previously determined by experts. In a few cases duplicates were sent to experts for confirmation. Voucher material is deposited in BHCB herbarium from Universidade Federal de Minas Gerais, Brazil. Abbreviation of authors’ names was based on IPNI (www.ipni.org). Previously known distribution of taxa was compiled from literature, especially floras and taxonomic treatments. For each new record we provide examined material, comments about previously known distribution and taxonomic notes.

## Taxon treatments

### Alsophila salvinii

Hook. 1866

urn:lsid:ipni.org:names:17018560-1

**Figure 1.**
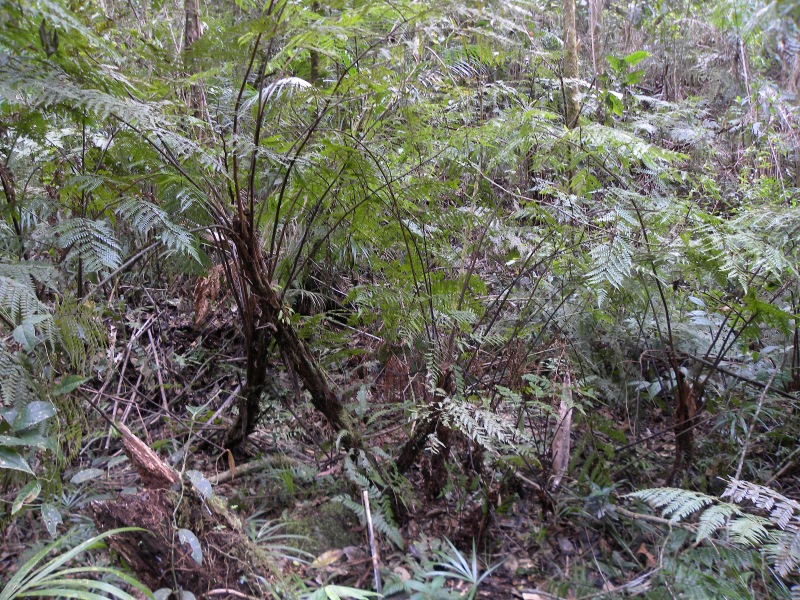
*Alsophila salvinii* Hook. (Cyatheaceae). Habit.

**Figure 2.**
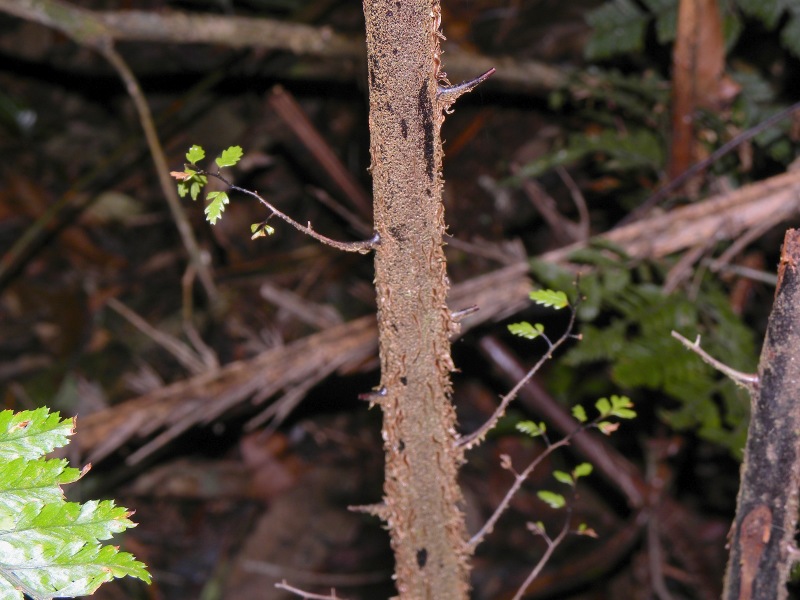
*Alsophila salvinii* Hook. (Cyatheaceae). Detail of aphlebiae at petiole base.

Cyatheaceae
Alsophila salvinii Hook., Syn. Fil. 36. 1866. Type: Guatemala, *Salvin & Godman s.n.* (K). Figs 1, 2.

#### Materials

- **Type status:** Other material. **Occurrence:** catalogNumber: BHCB 99175; recordNumber: A. Salino 11032; recordedBy: A. Salino et al.; **Taxon:** taxonID: urn:lsid:ipni.org:names:17018560-1; scientificName: *Alsophila salvinii* Hook.; kingdom: Plantae; class: Polypodiopsida; order: Cyatheales; family: Cyatheaceae; genus: Alsophila; specificEpithet: salvinii; scientificNameAuthorship: Hook.; **Location:** continent: South America; country: Brazil; countryCode: BR; stateProvince: Minas Gerais; municipality: Simonésia; locality: RPPN Mata do Sossego; verbatimElevation: 1150-1600 m; minimumElevationInMeters: 1150; maximumElevationInMeters: 1600; verbatimCoordinates: 20°04'02.0"S, 42°04'40.4"W; verbatimLatitude: 20°04'02.0"S; verbatimLongitude: 42°04'40.4"W; decimalLatitude: -20.067222; decimalLongitude: -42.077889; geodeticDatum: WGS84; **Identification:** identifiedBy: A. Salino; dateIdentified: 2007-11; **Event:** eventDate: 2006-05-20; year: 2006; month: 5; day: 20; **Record Level:** type: specimen; language: Portuguese; collectionCode: BHCB
- **Type status:** Other material. **Occurrence:** catalogNumber: BHCB 100000; recordNumber: A. Salino 11185; recordedBy: A. Salino et al.; **Taxon:** taxonID: urn:lsid:ipni.org:names:17018560-1; scientificName: *Alsophila salvinii* Hook.; kingdom: Plantae; class: Polypodiopsida; order: Cyatheales; family: Cyatheaceae; genus: Alsophila; specificEpithet: salvinii; scientificNameAuthorship: Hook.; **Location:** continent: South America; country: Brazil; countryCode: BR; stateProvince: Minas Gerais; municipality: Simonésia; locality: RPPN Mata do Sossego; verbatimElevation: 1150-1600 m; minimumElevationInMeters: 1150; maximumElevationInMeters: 1600; verbatimCoordinates: 20°04'18.7"S, 42°04'13.1"W; verbatimLatitude: 20°04'18.7"S; verbatimLongitude: 42°04'13.1"W; decimalLatitude: -20.071861; decimalLongitude: -42.070306; geodeticDatum: WGS84; **Identification:** identifiedBy: A. Salino; dateIdentified: 2006-06; **Event:** eventDate: 2006-05-24; year: 2006; month: 5; day: 24; **Record Level:** type: specimen; language: Portuguese; collectionCode: BHCB
- **Type status:** Other material. **Occurrence:** catalogNumber: BHCB 99330; recordNumber: A. Salino 11187; recordedBy: A. Salino et al.; **Taxon:** taxonID: urn:lsid:ipni.org:names:17018560-1; scientificName: *Alsophila salvinii* Hook.; kingdom: Plantae; class: Polypodiopsida; order: Cyatheales; family: Cyatheaceae; genus: Alsophila; specificEpithet: salvinii; scientificNameAuthorship: Hook.; **Location:** continent: South America; country: Brazil; countryCode: BR; stateProvince: Minas Gerais; municipality: Simonésia; locality: RPPN Mata do Sossego; verbatimElevation: 1150-1600 m; minimumElevationInMeters: 1150; maximumElevationInMeters: 1600; verbatimCoordinates: 20°04'18.7"S, 42°04'13.1"W; verbatimLatitude: 20°04'18.7"S; verbatimLongitude: 42°04'13.1"W; decimalLatitude: -20.071861; decimalLongitude: -42.070306; geodeticDatum: WGS84; **Identification:** identifiedBy: A. Salino; dateIdentified: 2007-11; **Event:** eventDate: 2006-05-24; year: 2006; month: 5; day: 24; **Record Level:** type: specimen; language: Portuguese; collectionCode: BHCB
- **Type status:** Other material. **Occurrence:** catalogNumber: BHCB 166827; recordNumber: T.E. Almeida 3338; recordedBy: T.E. Almeida et al.; **Taxon:** taxonID: urn:lsid:ipni.org:names:17018560-1; scientificName: *Alsophila salvinii* Hook.; kingdom: Plantae; class: Polypodiopsida; order: Cyatheales; family: Cyatheaceae; genus: Alsophila; specificEpithet: salvinii; scientificNameAuthorship: Hook.; **Location:** continent: South America; country: Brazil; countryCode: BR; stateProvince: Minas Gerais; municipality: Simonésia; locality: RPPN Mata do Sossego; verbatimElevation: 1183 m; minimumElevationInMeters: 1183; verbatimCoordinates: ﻿20°04'11"S, 42°04'18"W; verbatimLatitude: ﻿20°04'11"S; verbatimLongitude: 42°04'18"W; decimalLatitude: -20.069722; decimalLongitude: -42.071667; geodeticDatum: WGS84; **Identification:** identifiedBy: A. Salino; dateIdentified: 2013-11-17; **Event:** eventDate: 2013-11-17; year: 2013; month: 11; day: 17; **Record Level:** type: specimen; language: Portuguese; collectionCode: BHCB

#### Distribution

**Figure 3.**
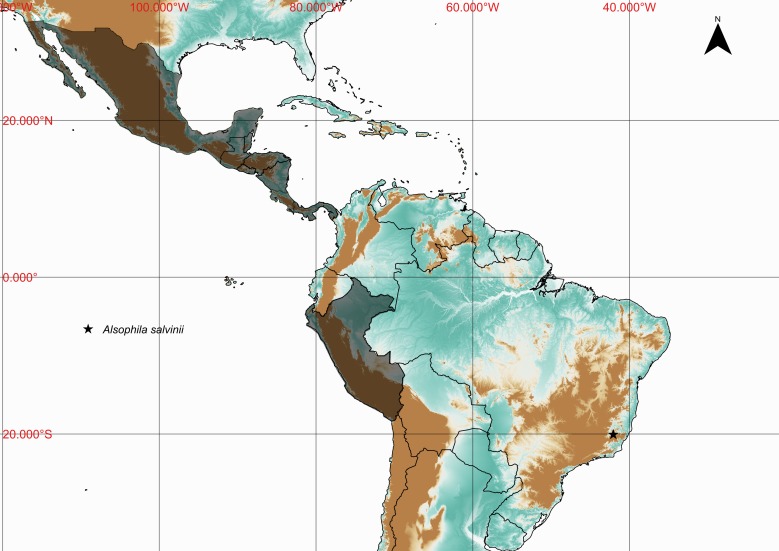
Distribution map of *Alsophila salvinii*, showing previously known distribution (shaded countries) and new record (star).

Previously known distribution: Belize, Costa Rica, El Salvador, Guatemala, Honduras, Mexico, Nicaragua, Panama and Peru (Moran 1995c, Smith et al. 2005). Fig. 3.

#### Ecology

Occurs as terrestrial in a fragment of Atlantic Rainforest.

#### Taxon discussion

This species can be recognized by petioles without conspicuous spines and with several pairs of aphlebiae toward the petiole bases (Moran 1995c, Conant 1983). Fig. 2​.

### Campyloneurum costatum

(Kunze) C.Presl 1836

urn:lsid:ipni.org:names:17063070-1

**Figure 4.**
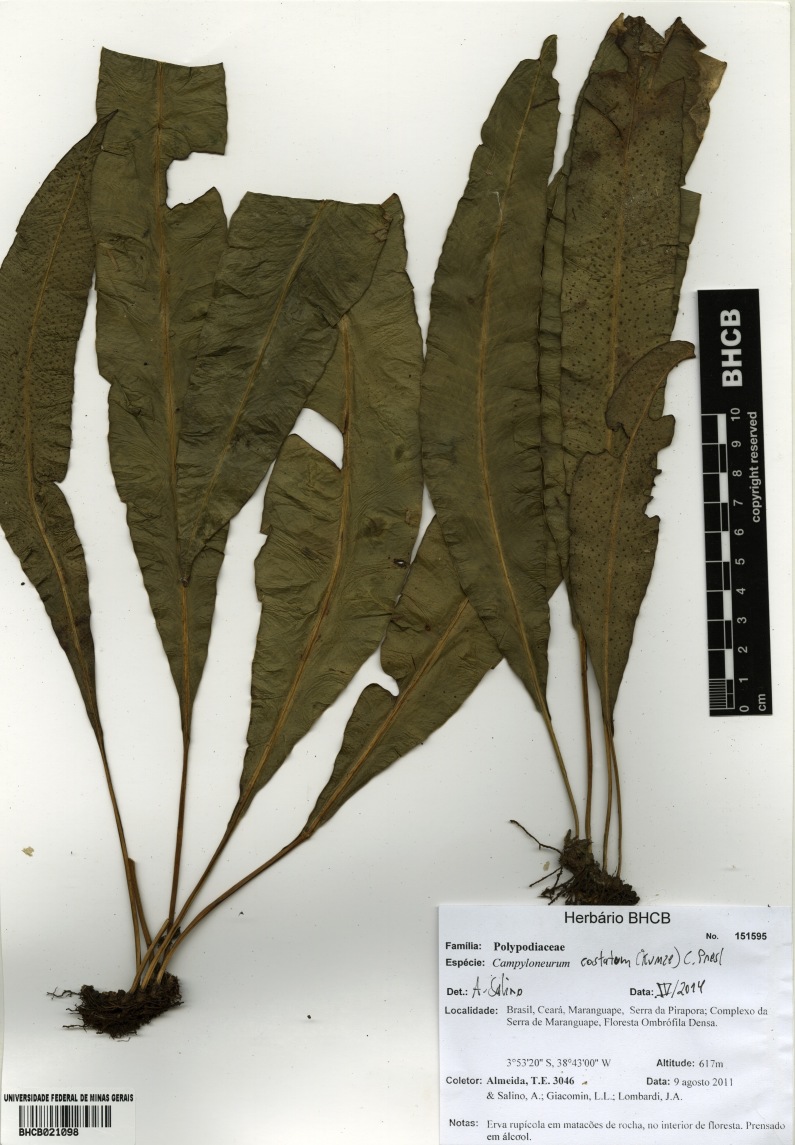
*Campyloneurum costatum* (Kunze) C.Presl (Polypodiaceae).

Polypodiaceae
Campyloneurum costatum (Kunze) C.Presl, Tent. Pterid. 190. 1836. *Polypodium costatum* Kunze, Linnaea 9: 38. 1834. Type: Cuba, *Poeppigs.n.* (LZ). Fig. 4.

#### Materials

- **Type status:** Other material. **Occurrence:** catalogNumber: BHCB 151595; recordNumber: T.E. Almeida 3046; recordedBy: T.E. Almeida et al.; **Taxon:** taxonID: urn:lsid:ipni.org:names:17063070-1; scientificName: *Campyloneurum costatum* (Kunze) C.Presl; kingdom: Plantae; class: Polypodiopsida; family: Polypodiaceae; genus: Campyloneurum; specificEpithet: costatum; scientificNameAuthorship: (Kunze) C.Presl; **Location:** continent: South America; country: Brazil; countryCode: BR; stateProvince: Ceará; municipality: Maranguape; locality: Serra da Pirapora, complexo da Serra de Maranguape; verbatimElevation: 617 m; minimumElevationInMeters: 617; verbatimCoordinates: 03°53'20.0"S, 38°43'00.0"W; verbatimLatitude: 03°53'20.0"S; verbatimLongitude: 38°43'00.0"W; decimalLatitude: -3.888889; decimalLongitude: -38.716667; geodeticDatum: WGS84; **Identification:** identifiedBy: A. Salino; dateIdentified: 2014-04-02; **Event:** eventDate: 2008-08-09; year: 2008; month: 8; day: 9; **Record Level:** type: specimen; language: Portuguese; collectionCode: BHCB

#### Distribution

**Figure 5.**
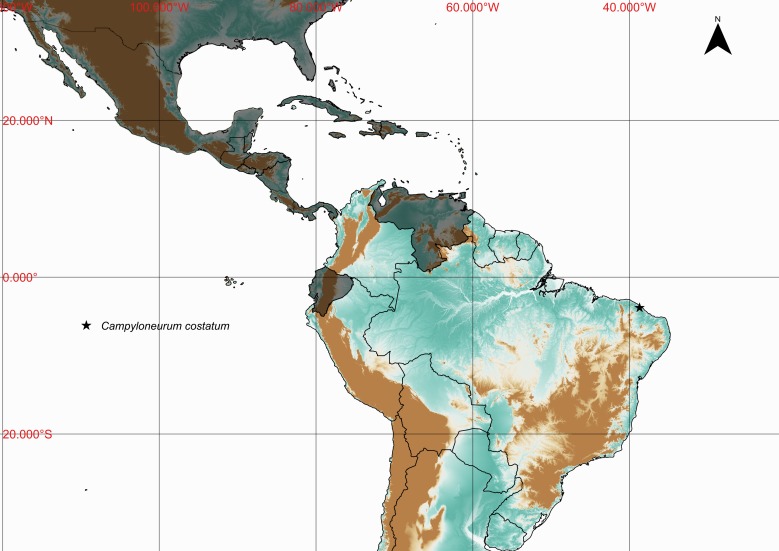
Distribution map of *Campyloneurum costatum*, showing previously known distribution (shaded countries) and new record (star).

Previously known distribution: Southern United States to Panama, Greater Antilles, Trinidad, Venezuela and Ecuador (León 1993). Fig. 5.

#### Ecology

Occurs as terrestrial or low epiphyte in montane wet forests.

#### Taxon discussion

This species can be recognized by the lanceolate or elliptical-lanceolate leaves, with inconspicuous or slightly prominulous veins (León 1993). The closest species is *Campyloneurum xalapense* Fée, from which it differs by the leave shape (León 1993).

### Cyathea subincisa

(Kunze) Domin 1929

urn:lsid:ipni.org:names:17314710-1

**Figure 6.**
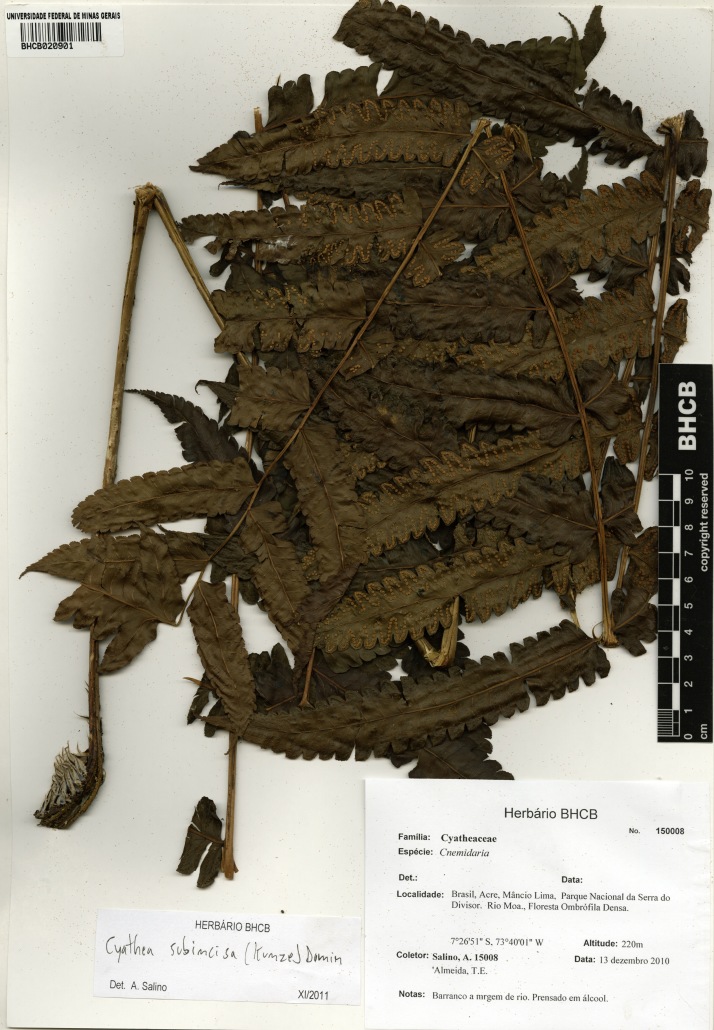
*Cyathea subincisa* (Kunze) Domin (Cyatheaceae).

Cyatheaceae
Cyathea subincisa (Kunze) Domin, Pteridophyta 264. 1929. *Hemitelia subincisa* Kunze, Bot. Zeit. 2: 296. 1844. Type: Peru, *Poeppig 221* (PR or PRC). Fig. 6.

#### Materials

- **Type status:** Other material. **Occurrence:** catalogNumber: BHCB 150008; recordNumber: A. Salino 15008; recordedBy: A. Salino & T.E. Almeida; **Taxon:** taxonID: urn:lsid:ipni.org:names:17314710-1; scientificName: *Cyathea subincisa* (Kunze) Domin; kingdom: Plantae; class: Polypodiopsida; order: Cyatheales; family: Cyatheaceae; genus: Cyathea; specificEpithet: subincisa; scientificNameAuthorship: (Kunze) Domin; **Location:** continent: South America; country: Brazil; countryCode: BR; stateProvince: Acre; municipality: Mâncio Lima; locality: Parque Nacional Serra do Divisor, Rio Môa,; verbatimElevation: 220 m; minimumElevationInMeters: 220; verbatimCoordinates: 07°26'51"S, 73°40'01"W; verbatimLatitude: 07°26'51"S; verbatimLongitude: 73°40'01"W; decimalLatitude: -7.4475; decimalLongitude: -73.666944; geodeticDatum: WGS84; **Identification:** identifiedBy: A. Salino; dateIdentified: 2011-10; **Event:** eventDate: 2010-12-13; year: 2010; month: 12; day: 13; **Record Level:** type: specimen; language: Portuguese; collectionCode: BHCB

#### Distribution

**Figure 7.**
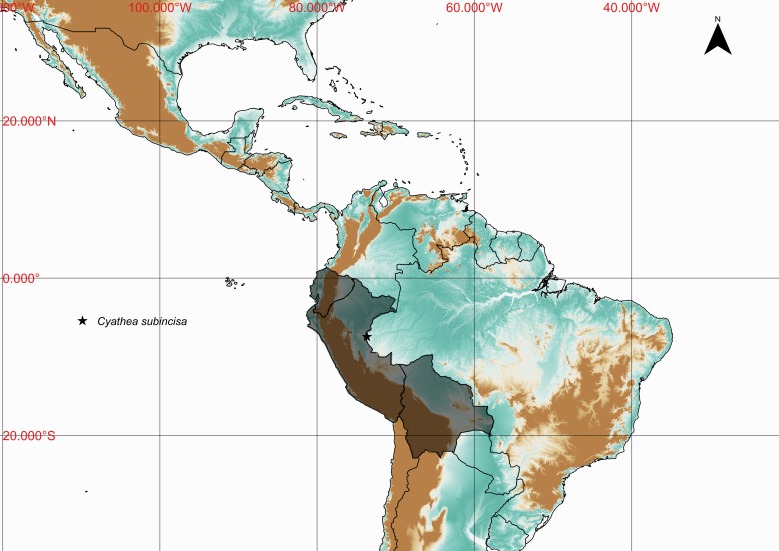
Distribution map of *Cyathea subincisa* showing previously known distribution (shaded countries) and new record (star).

Previously known distribution: Bolivia, Ecuador and Peru (Lehnert 2011). Fig. 7.

#### Ecology

Occurs as rupestrial in rocky cliffs at river margins.

#### Taxon discussion

This species is characterized by the conform or subconform apical pinnae, sori medial to supramedial and petioles smooth (Stolze 1974). The closest species is *Cyathea consimilis* (Stolze) Lehnert (Stolze 1974) from which *C. subincisa* can be distinguished by the smooth or rarely tuberculate petiole (spine or muricate in *C. consimilis*).

### Cyclodium trianae

(Mett.) A.R.Sm. 1986

urn:lsid:ipni.org:names:275528-2

**Figure 8.**
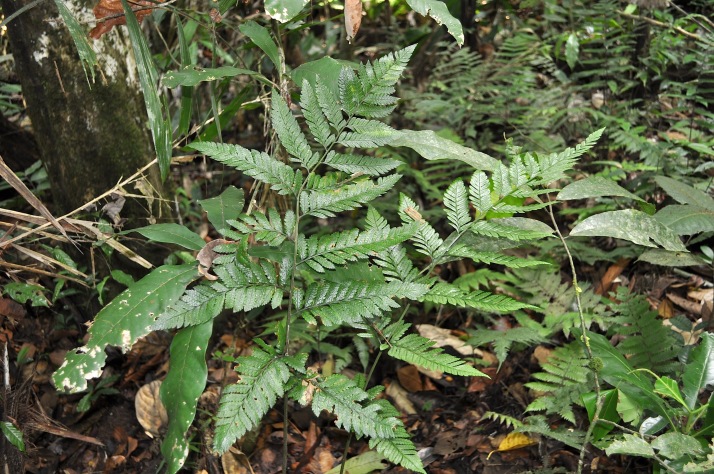
*Cyclodium trianae* (Mett.) A.R.Sm. (Dryopteridaceae).

Dryopteridaceae
Cyclodium trianae (Mett.) A.R.Sm., Amer. Fern J. 76(2): 56-98. 1986. *Aspidium trianae* Mett., Ann. Sci. Nat. Bot., V. 2: 243. 1864. Type: Colombia, *Triana 32* (B). Fig. 8.

#### Materials

- **Type status:** Other material. **Occurrence:** catalogNumber: BHCB 144684; recordNumber: T.E. Almeida 2561; recordedBy: T.E. Almeida & A. Salino; **Taxon:** taxonID: urn:lsid:ipni.org:names:275528-2; scientificName: *Cyclodium trianae* (Mett.) A.R.Sm.; kingdom: Plantae; class: Polypodiopsida; order: Polypodiales; family: Dryopteridaceae; genus: Cyclodium; specificEpithet: trianae; scientificNameAuthorship: (Mett.) A.R.Sm.; **Location:** continent: South America; country: Brazil; countryCode: BR; stateProvince: Acre; municipality: Cruzeiro do Sul; locality: Comunidade Santa Luzia, 45 km from Cruzeiro do Sul on BR-364; verbatimElevation: 276 m; minimumElevationInMeters: 276; verbatimCoordinates: 07°53'45"S, 72°24'30"W; verbatimLatitude: 07°53'45"S; verbatimLongitude: 72°24'30"W; decimalLatitude: -7.895833; decimalLongitude: -72.408333; geodeticDatum: WGS84; **Identification:** identifiedBy: A. Salino & A.R. Smith; dateIdentified: 2011-12-06; **Event:** eventDate: 2010-12-10; year: 2010; month: 12; day: 10; **Record Level:** type: specimen; language: Portuguese; collectionCode: BHCB

#### Distribution

**Figure 9.**
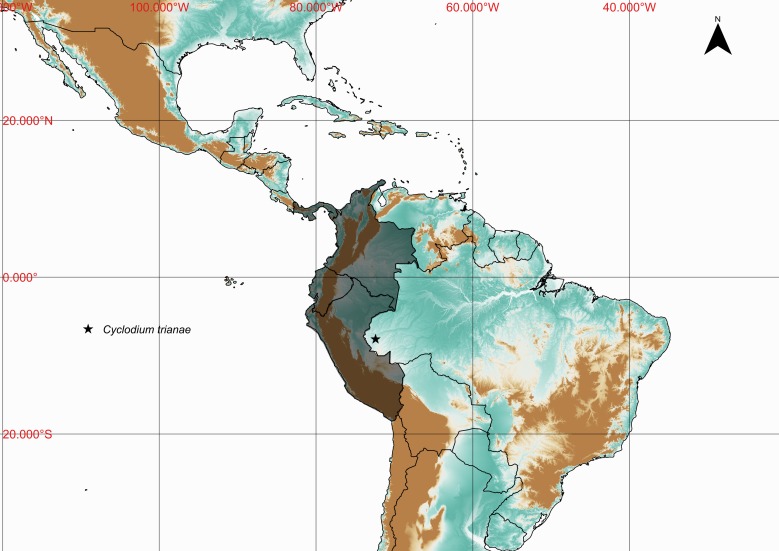
Distribution map of *Cyclodium trianae* showing previously known distribution (shaded countries) and new record (star).

Previously known distribution: Colombia, Ecuador, Panama and Peru (Smith 1986). Fig. 9.

#### Ecology

Occurs as terrestrial at low elevations, usually below 500 m, at eastern side of Andes.

#### Taxon discussion

In his revision of Neotropical *Cyclodium*, Smith (1986) cited that this species and *Cyclodium seemannii* (Hook.) A.R.Sm. can be distinguished from all other species of the genus by the round-reniform indusia. *Cyclodium trianae* can be readily separated from *C. seemannii* by the lack of sessile and globose glands in the lamina abaxially (Smith 1986).

### Elaphoglossum stenophyllum

(Sodiro) Diels 1899

urn:lsid:ipni.org:names:17105590-1

**Figure 10.**
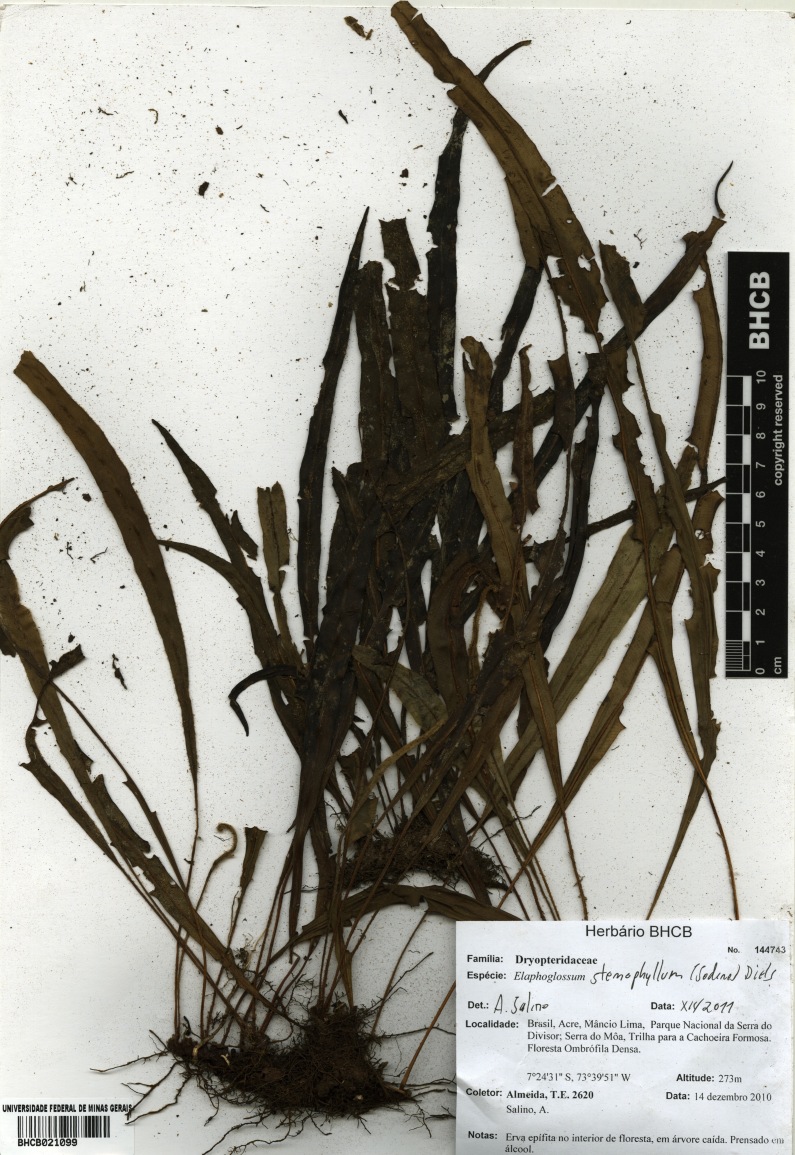
*Elaphoglossum stenophyllum* (Sodiro) Diels (Dryopteridaceae).

Dryopteridaceae
Elaphoglossum stenophyllum (Sodiro) Diels, Nat. Pfanzenfam. 1(4): 333. 1899. *Acrostichum stenophyllum* Sodiro, Crypt. Vasc. Quit. 468. 1893. Type: Ecuador, *Sodiros.n.* (US). Fig. 10.

#### Materials

- **Type status:** Other material. **Occurrence:** catalogNumber: BHCB 144743; recordNumber: T.E. Almeida 2620; recordedBy: T.E. Almeida & A. Salino; **Taxon:** taxonID: urn:lsid:ipni.org:names:17105590-1; scientificName: *Elaphoglossum stenophyllum* (Sodiro) Diels; kingdom: Plantae; class: Polypodiopsida; order: Polypodiales; family: Dryopteridaceae; genus: Elaphoglossum; specificEpithet: stenophyllum; scientificNameAuthorship: (Sodiro) Diels; **Location:** continent: South America; country: Brazil; countryCode: BR; stateProvince: Acre; municipality: Mâncio Lima; locality: Parque Nacional da Serra do Divisor, Serra do Môa, trail to Cachoeira Formosa; verbatimElevation: 273 m; minimumElevationInMeters: 273; verbatimCoordinates: 07°24'31"S, 73°39'51"W; verbatimLatitude: 07°24'31"S; verbatimLongitude: 73°39'51"W; decimalLatitude: -7.408611; decimalLongitude: -73.664167; geodeticDatum: WGS84; **Identification:** identifiedBy: A. Salino; dateIdentified: 2011-12; **Event:** eventDate: 2010-12-14; year: 2010; month: 12; day: 14; **Record Level:** type: specimen; language: Portuguese; collectionCode: BHCB

#### Distribution

**Figure 11.**
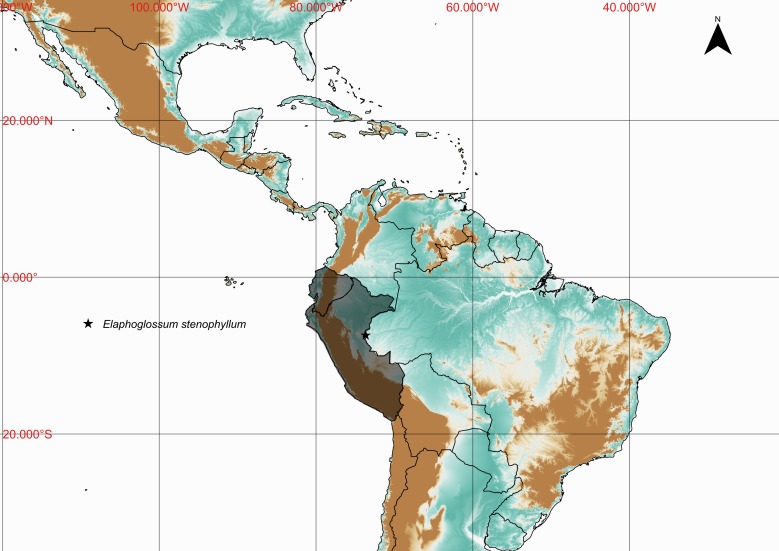
Distribution map of *Elaphoglossum stenophyllum*, showing previously known distribution (shaded countries) and new record (star).

Previously known distribution: Ecuador and Peru (Tryon et al. 1991). Fig. 11.

#### Ecology

Occurs as epiphyte or terrestrial in wet forest.

#### Taxon discussion

According to Tryon et al. (1991), this species resembles *Elaphoglossum tectum* (Willd.) T.Moore but differs from it by having glandular dots in the abaxial surface instead of stellate trichomes.

### Hypoderris brauniana

(H.Karst.) F.G.Wang & Christenh. 2014

urn:lsid:ipni.org:names:77140308-1

**Figure 12.**
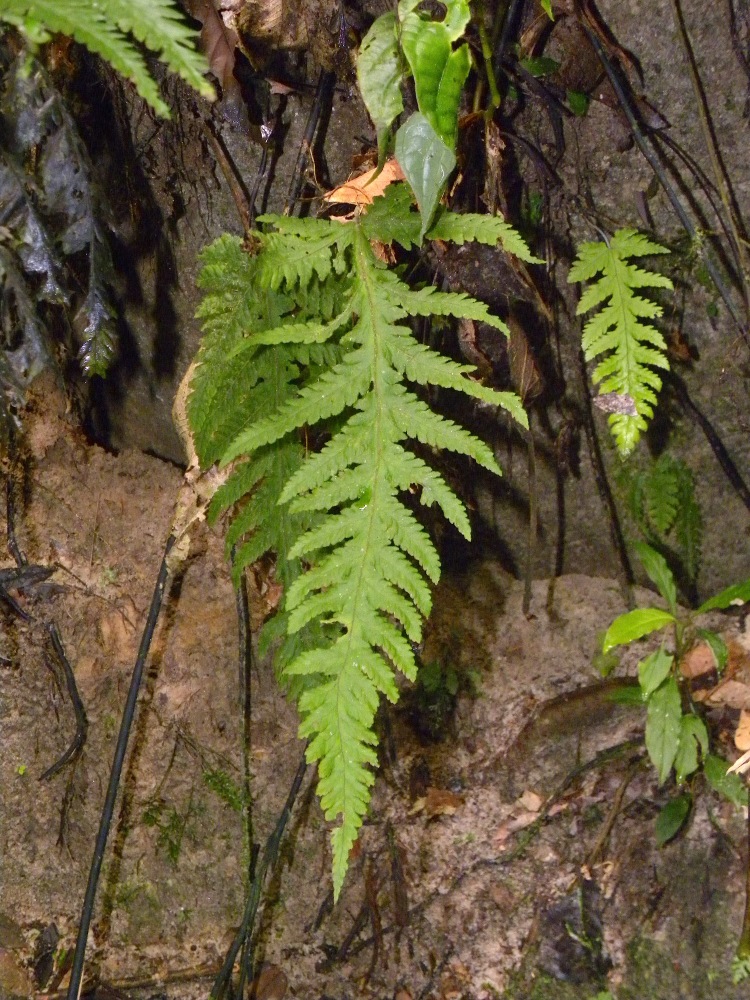
*Hypoderris brauninana* (H.Karst.) F.G.Wang & Christenh. (Tectariaceae).

Tectariaceae
Hypoderris brauniana (H.Karst.) F.G.Wang & Christenh., Phytotaxa 164(1): 12. 2014. *Aspidium braunianum* Karsten, Fl. Columb. 1: 63. 1859. Type: Colombia, *Karstens.n.* (W?). Fig. 12.

#### Materials

- **Type status:** Other material. **Occurrence:** catalogNumber: BHCB 144701; recordNumber: T.E. Almeida 2578; recordedBy: T.E. Almeida & A. Salino; **Taxon:** taxonID: urn:lsid:ipni.org:names:77140308-1; scientificName: *Hypoderris brauniana* (H.Karst.) F.G.Wang & Christenh.; kingdom: Plantae; class: Polypodiopsida; order: Polypodiales; family: Dryopteridaceae; genus: Hypoderris; specificEpithet: brauniana; scientificNameAuthorship: (H.Karst.) F.G.Wang & Christenh.; **Location:** continent: South America; country: Brazil; countryCode: BR; stateProvince: Acre; municipality: Mâncio Lima; locality: Parque Nacional da Serra do Divisor, Serra do Môa, trail from Igarapé do Amor to Cachoeira da Estátua; verbatimElevation: 218 m; minimumElevationInMeters: 218; verbatimCoordinates: 07°26'51"S, 73°40'01"W; verbatimLatitude: 07°26'51"S; verbatimLongitude: 73°40'01"W; decimalLatitude: -7.4475; decimalLongitude: -73.666944; geodeticDatum: WGS84; **Identification:** identifiedBy: A. Salino & V.A.O. Dittrich; dateIdentified: 2010-12; **Event:** eventDate: 2010-12-13; year: 2010; month: 12; day: 13; **Record Level:** type: specimen; language: Portuguese; collectionCode: BHCB

#### Distribution

**Figure 13.**
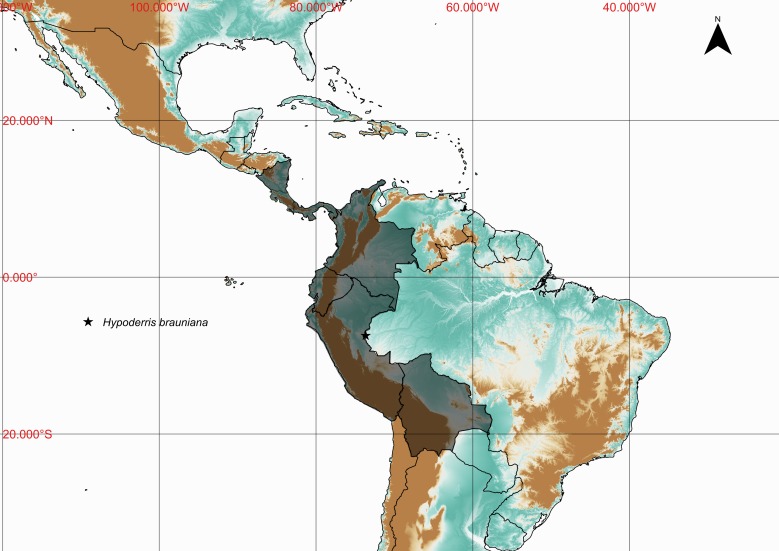
Distribution map of *Hypoderris brauniana*, showing previously known distribution (shaded countries) and new record (star).

Previously known distribution: Bolivia, Colombia, Costa Rica, Ecuador, Nicaragua, Panama and Peru (Moran 1995d). Fig. 13.

#### Ecology

Occurs as terrestrial in wet forests along small streams.

#### Taxon discussion

This species can be readily distinguished by the creeping rhizomes, free venation, 2-pinnatifid lamina, rachis ablate and tawny indument (Moran et al. 2014). The closest species are *Hypoderris brownii* J.Sm. and *H. nicotianifolia* (Baker) R.C.Moran, Labiak & J.Prado, which present reticulate veins (Moran et al. 2014).

### Pleopeltis stolzei

A.R.Sm. 2005

urn:lsid:ipni.org:names:77067049-1

**Figure 14.**
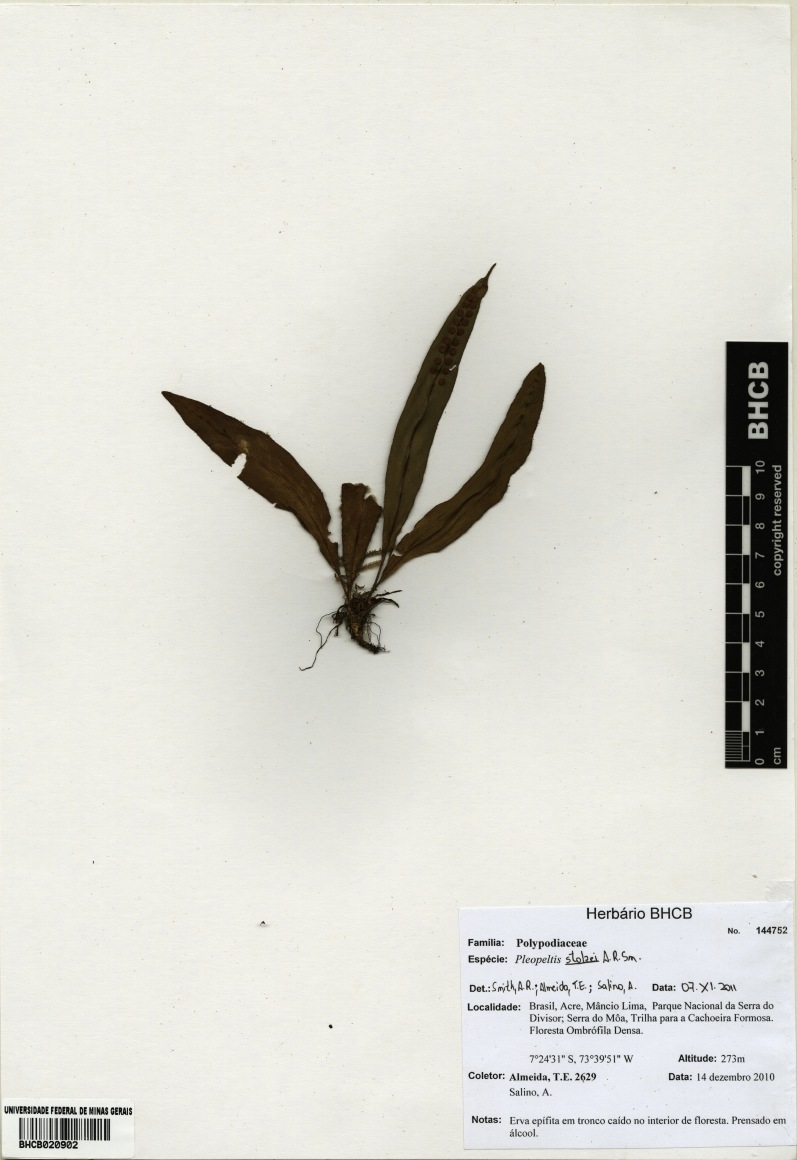
*Pleopeltis stolzei* A.R.Sm. (Polypodiaceae).

Polypodiaceae
Pleopeltis stolzei A.R.Sm., Candollea 60: 262. 2005. Pleopeltis macrocarpa var. laciniata Stolze, Fieldiana, Bot. 2, 32: 143. 1993. Type: Peru, *Moran & Fernández 3681* (USM). Fig. 14.

#### Materials

- **Type status:** Other material. **Occurrence:** catalogNumber: INPA 28165; recordNumber: Byron 304; recordedBy: Byron et J. Lima; **Taxon:** taxonID: urn:lsid:ipni.org:names:77067049-1; scientificName: *Pleopeltis stolzei* A.R.Sm.; kingdom: Plantae; class: Polypodiopsida; order: Polypodiales; family: Polypodiaceae; genus: Pleopeltis; specificEpithet: stolzei; scientificNameAuthorship: A.R.Sm.; **Location:** continent: South America; country: Brazil; countryCode: BR; stateProvince: Amazonas; municipality: Barreirinha; locality: Rio Auatí Paraná, igarapé Josefina; verbatimLocality: 273; **Identification:** identifiedBy: T.E. Almeida; dateIdentified: 2015-01-05; **Event:** eventDate: 1970-04-14; year: 1970; month: 4; day: 14; **Record Level:** type: specimen; language: Portuguese; collectionCode: INPA
- **Type status:** Other material. **Occurrence:** catalogNumber: BHCB 144752; recordNumber: T.E. Almeida 2629; recordedBy: T.E. Almeida & A. Salino; **Taxon:** taxonID: urn:lsid:ipni.org:names:77067049-1; scientificName: *Pleopeltis stolzei* A.R.Sm.; kingdom: Plantae; class: Polypodiopsida; order: Polypodiales; family: Polypodiaceae; genus: Pleopeltis; specificEpithet: stolzei; scientificNameAuthorship: A.R.Sm.; **Location:** continent: South America; country: Brazil; countryCode: BR; stateProvince: Acre; municipality: Mâncio Lima; locality: Parque Nacional da Serra do Divisor, Serra do Môa, trail to Cachoeira Formosa; verbatimLocality: 273; verbatimElevation: 273 m; minimumElevationInMeters: 273; verbatimCoordinates: 07°24'31"S, 73°39'51"W; verbatimLatitude: 07°24'31"S; verbatimLongitude: 73°39'51"W; decimalLatitude: -7.408611; decimalLongitude: -73.664167; geodeticDatum: WGS84; **Identification:** identifiedBy: T.E. Almeida & A.R. Smith; **Event:** eventDate: 2010-12-14; year: 2010; month: 12; day: 14; **Record Level:** type: specimen; language: Portuguese; collectionCode: BHCB

#### Distribution

**Figure 15.**
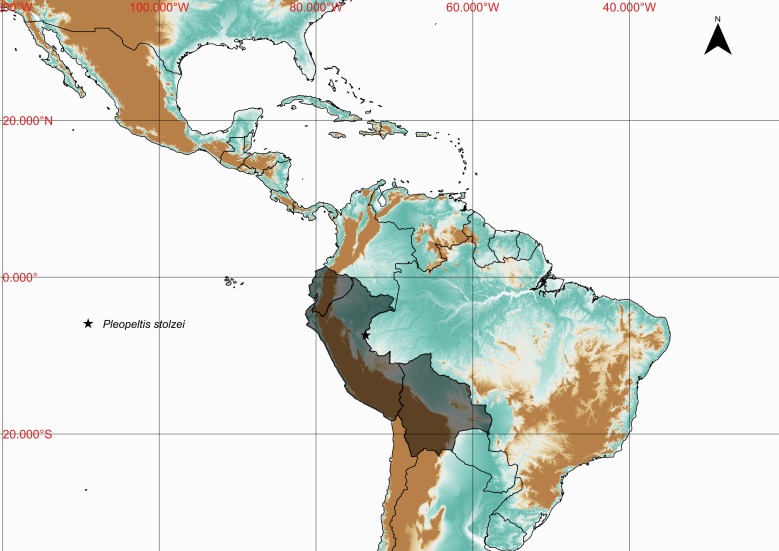
Distribution map of *Pleopeltis stolzei*, showing previously known distribution (shaded countries) and new record (star).

Previously known distribution: Bolivia, Ecuador and Peru (Kessler and Smith 2005). Fig. 15.

#### Ecology

Occurs as epiphyte in wet forest.

#### Taxon discussion

This species can be distinguished from *Pleopeltis macrocarpa* (Bory ex Willd) Kaulf., species from which it was previously recognized as the variety Pleopeltis macrocarpa var. laciniata Stolze, by the laminar scales concolorous with laciniate margins (Tryon et al. 1993). It can also be recognized by the larger lamina and also by its shape, with broad to narrow-cuneate base (Tryon et al. 1993).

### Polypodium flagellare

Christ 1896

urn:lsid:ipni.org:names:206959-2

**Figure 16.**
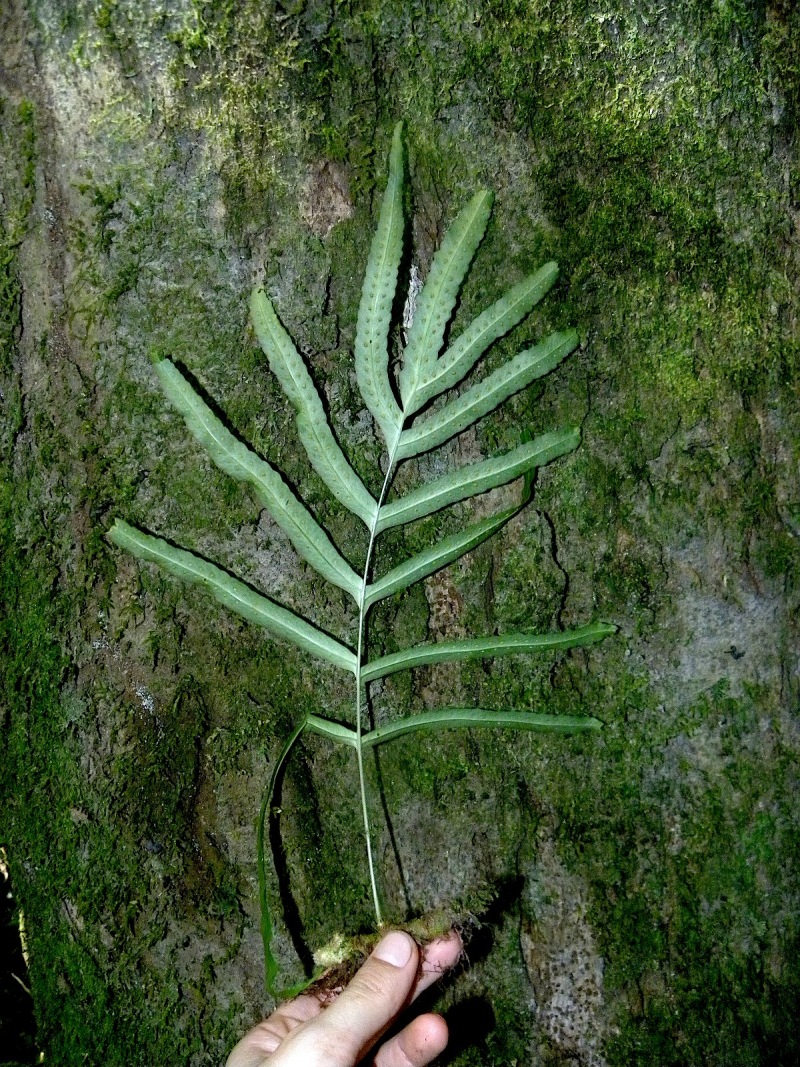
*Polypodium flagellare* Christ (Polypodiaceae).

Polypodiaceae
Polypodium flagellare Christ, Bull. Herb. Boissier 4(10): 660. 1896. Type: Costa Rica, *Biolley 2671* (BR). Fig. 16.

#### Materials

- **Type status:** Other material. **Occurrence:** catalogNumber: BHCB 136570; recordNumber: T.E. Almeida 2219; recordedBy: T.E. Almeida et al.; **Taxon:** taxonID: urn:lsid:ipni.org:names:206959-2; scientificName: *Polypodium flagellare* Christ; kingdom: Plantae; class: Polypodiopsida; order: Polypodiales; family: Polypodiaceae; genus: Polypodium; specificEpithet: flagellare; scientificNameAuthorship: Christ; **Location:** continent: South America; country: Brazil; countryCode: BR; stateProvince: Pará; municipality: Canaã dos Carajás; locality: Floresta Nacional de Carajás, Serra Sul; verbatimElevation: 611 m; minimumElevationInMeters: 611; verbatimCoordinates: 06°22'44"S, 50°22'38"W; verbatimLatitude: 06°22'44"S; verbatimLongitude: 50°22'38"W; decimalLatitude: -6.378889; decimalLongitude: -50.377222; geodeticDatum: WGS84; **Identification:** identifiedBy: A.R. Smith; dateIdentified: 2010-03; **Event:** eventDate: 2010-02-16; year: 2010; month: 2; day: 16; **Record Level:** type: specimen; language: Portuguese; collectionCode: BHCB
- **Type status:** Other material. **Occurrence:** catalogNumber: BHCB 140427; recordNumber: D.T. Souza 1129; recordedBy: D.T. Souza et al.; **Taxon:** taxonID: urn:lsid:ipni.org:names:206959-2; scientificName: *Polypodium flagellare* Christ; kingdom: Plantae; class: Polypodiopsida; order: Polypodiales; family: Polypodiaceae; genus: Polypodium; specificEpithet: flagellare; scientificNameAuthorship: Christ; **Location:** continent: South America; country: Brazil; countryCode: BR; stateProvince: Pará; municipality: Canaã dos Carajás; locality: Floresta Nacional de Carajás, Serra Sul; verbatimElevation: 677 m; minimumElevationInMeters: 677; verbatimCoordinates: 06°20'41"S, 50°27'05"W; verbatimLatitude: 06°20'41"S; verbatimLongitude: 50°27'05"W; decimalLatitude: -6.344722; decimalLongitude: -50.451389; geodeticDatum: WGS84; **Identification:** identifiedBy: T.E. Almeida; dateIdentified: 2010-06; **Event:** eventDate: 2010-05-21; year: 2010; month: 5; day: 21; **Record Level:** type: specimen; language: Portuguese; collectionCode: BHCB

#### Distribution

**Figure 17.**
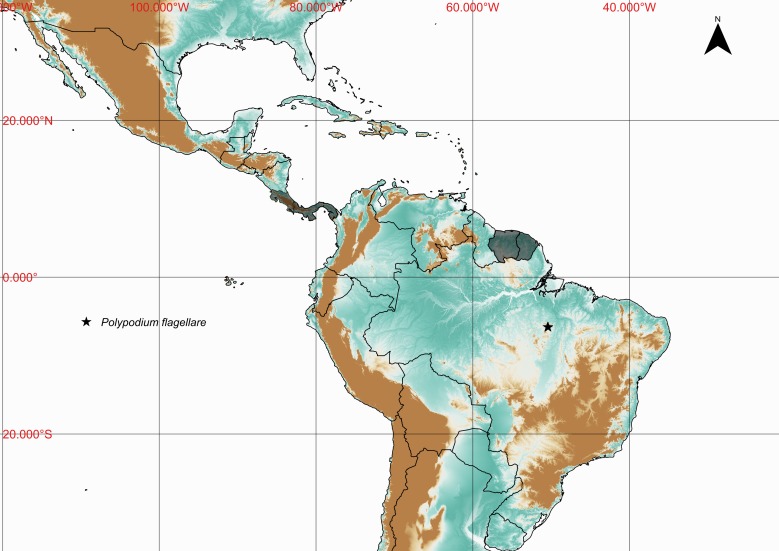
Distribution map of *Polypodium flagellare*, showing previously known distribution (shaded countries) and new record (star).

Previously known distribution: Costa Rica, French Guiana, Panama and Suriname (Moran 1995c). Fig. 17.

#### Ecology

Occurs as epiphyte in montane rainforest.

#### Taxon discussion

This species can be recognized by the pendent leaves, one row of areoles between costa and margin and pinnae sessile to adnate (Moran 1995c). Affinities of *P. flagellare* as well as the relationships among *Polypodium* *s.s.* species are still uncertain (Tejero-Díez 2005).

### Tectaria heracleifolia

(Willd.) Underw. 1906

urn:lsid:ipni.org:names:17360470-1

**Figure 18.**
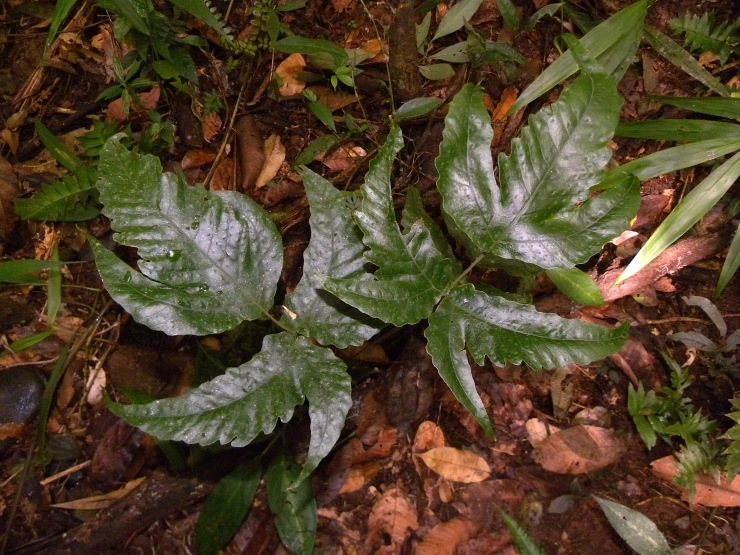
*Tectaria heracleifolia* (Willd.) Underwood. (Tectariaceae).

Tectariaceae
Tectaria heracleifolia (Willd.) Underw., Bull. Torrey Bot. Club 33: 200. 1906. *Aspidium heracleifolium* Willd., Sp. Pl. 5: 217. 1810. Type: Plumier, Traité Foug. Amér. T. 126. 1705. Fig. 18.

#### Materials

- **Type status:** Other material. **Occurrence:** catalogNumber: BHCB ﻿136597; recordNumber: T.E. Almeida 2246; recordedBy: T.E. Almeida et al.; **Taxon:** taxonID: urn:lsid:ipni.org:names:17360470-1; scientificName: *Tectaria heracleifolia* (Willd.) Underw.; kingdom: Plantae; class: Polypodiopsida; order: Polypodiales; family: Tectariaceae; genus: Tectaria; specificEpithet: heracleifolia; scientificNameAuthorship: (Willd.) Underw.; **Location:** continent: South America; country: Brazil; countryCode: BR; stateProvince: Pará; municipality: Canaã dos Carajás; locality: Floresta Nacional de Carajás, Serra Sul; verbatimElevation: 530 m; minimumElevationInMeters: 530; verbatimCoordinates: 06°19'58"S, 50°24'46"W; verbatimLatitude: 06°19'58"S; verbatimLongitude: 50°24'46"W; decimalLatitude: -6.332778; decimalLongitude: -50.412778; geodeticDatum: WGS84; **Identification:** identifiedBy: A. Salino; **Event:** eventDate: 2010-02-17; year: 2010; month: 2; day: 17; **Record Level:** type: specimen; language: Portuguese; collectionCode: BHCB
- **Type status:** Other material. **Occurrence:** catalogNumber: BHCB ﻿139524; recordNumber: T.E. Almeida 2332; recordedBy: T.E. Almeida et al.; **Taxon:** taxonID: urn:lsid:ipni.org:names:17360470-1; scientificName: *Tectaria heracleifolia* (Willd.) Underw.; kingdom: Plantae; class: Polypodiopsida; order: Polypodiales; family: Tectariaceae; genus: Tectaria; specificEpithet: heracleifolia; scientificNameAuthorship: (Willd.) Underw.; **Location:** continent: South America; country: Brazil; countryCode: BR; stateProvince: Pará; municipality: Canaã dos Carajás; locality: Floresta Nacional de Carajás, Serra Sul, Córrego da Cachoeira; verbatimElevation: 377 m; minimumElevationInMeters: 377; verbatimCoordinates: 06°24'25"S, 50°14'57"W; verbatimLatitude: 06°24'25"S; verbatimLongitude: 50°14'57"W; decimalLatitude: -6.406944; decimalLongitude: -50.249167; geodeticDatum: WGS84; **Identification:** identifiedBy: T.E. Almeida & A. Salino; dateIdentified: 2010-05-17; **Event:** eventDate: 2010-04-27; year: 2010; month: 4; day: 27; **Record Level:** type: specimen; language: Portuguese; collectionCode: BHCB
- **Type status:** Other material. **Occurrence:** catalogNumber: BHCB ﻿139520; recordNumber: T.E. Almeida 2328; recordedBy: T.E. Almeida et al.; **Taxon:** taxonID: urn:lsid:ipni.org:names:17360470-1; scientificName: *Tectaria heracleifolia* (Willd.) Underw.; kingdom: Plantae; class: Polypodiopsida; order: Polypodiales; family: Tectariaceae; genus: Tectaria; specificEpithet: heracleifolia; scientificNameAuthorship: (Willd.) Underw.; **Location:** continent: South America; country: Brazil; countryCode: BR; stateProvince: Pará; municipality: Canaã dos Carajás; locality: Pará: Canaã dos Carajás, Floresta Nacional de Carajás, Serra Sul, Córrego da Cachoeira; verbatimElevation: 377 m; minimumElevationInMeters: 377; verbatimCoordinates: 06°24'25"S, 50°14'57"W; verbatimLatitude: 06°24'25"S; verbatimLongitude: 50°14'57"W; decimalLatitude: -6.406944; decimalLongitude: -50.249167; geodeticDatum: WGS84; **Identification:** identifiedBy: T.E. Almeida & A. Salino; dateIdentified: 2010-05-17; **Event:** eventDate: 2010-04-27; year: 2010; month: 4; day: 27; **Record Level:** type: specimen; language: Portuguese; collectionCode: BHCB

#### Distribution

**Figure 19.**
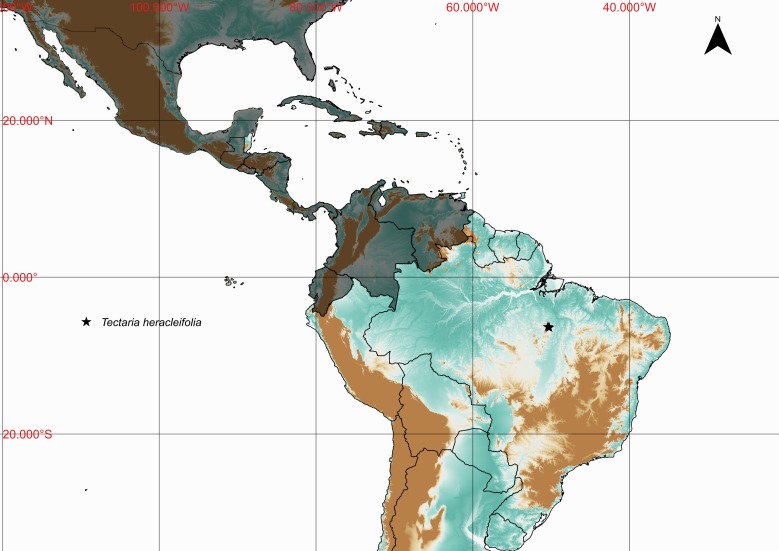
Distribution map of *Tectaria heracleifolia*, showing previously known distribution (shaded countries) and new record (star).

Known distribution: Antilles, Colombia, Costa Rica, Ecuador, El Salvador, Guatemala, Honduras, Mexico, Nicaragua, Panama, United States of America and Venezuela (Moran 1995b). Fig. 19.

#### Ecology

Occurs as terrestrial or rupestrial in montane wet and seasonal forests.

#### Taxon discussion

This is a very common species in Central America, also occurring in northern South America. It can be recognized by peltate indusia and entire pinnae or lobes (Moran 1995b). The closest species is *Tectaria incisa* Cav., from which *T. heracleifolia* can be distinguished by peltate indusia, cordiform bases of pinnae and apical segment and smaller number of pinnae (Moran 1995b).

### Thelypteris (Goniopteris) rolandii

(C.Chr.) R.M.Tryon 1967

urn:lsid:ipni.org:names:252305-2

**Figure 20.**
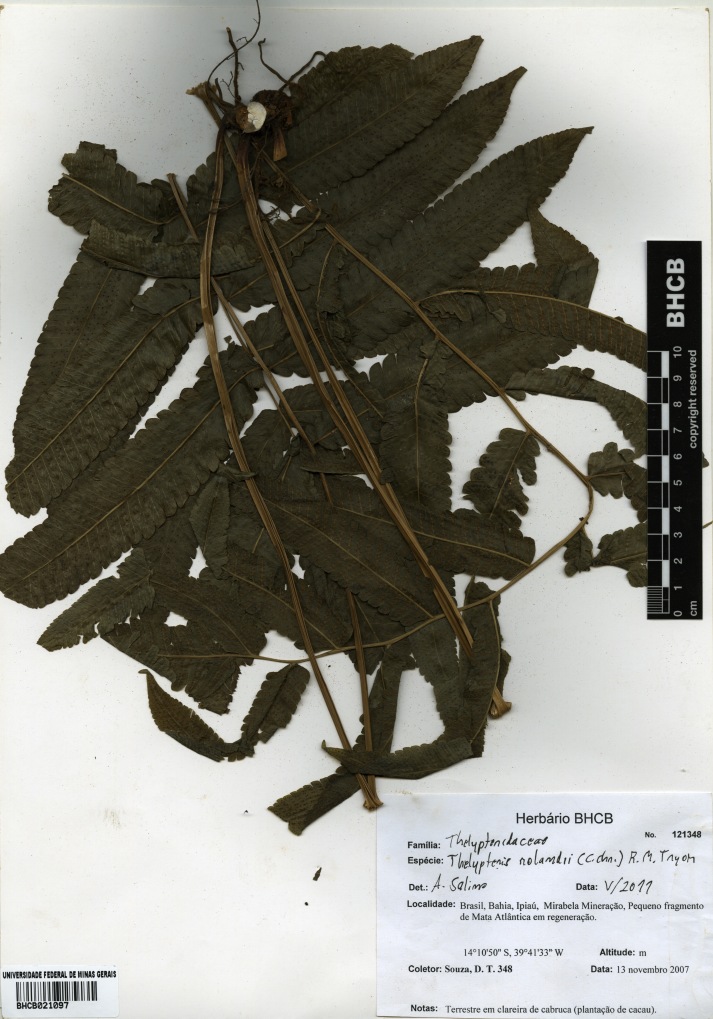
Thelypteris (Goniopteris) rolandii (C.Chr.) R.M.Tryon (Thelypteridaceae).

Thelypteridaceae
Thelypteris (Goniopteris) rolandii (C.Chr.) R.M.Tryon, Rhodora 69: 8. 1967. *Dryopteris rolandii* C.Chr., Kongel. Danske Vidensk. Selsk. Skr., Naturvidensk. Math. Afd. 7, 10: 258. 1913. Type: Ecuador, *Spruce 5718* (P). Fig. 20​.

#### Materials

- **Type status:** Other material. **Occurrence:** catalogNumber: BHCB 121348; recordNumber: D.T. Souza 348; recordedBy: D.T. Souza; **Taxon:** taxonID: urn:lsid:ipni.org:names:252305-2; scientificName: Thelypteris (Goniopteris) rolandii (C.Chr.) R.M.Tryon; kingdom: Plantae; class: Polypodiopsida; order: Polypodiales; family: Thelypteridaceae; genus: Thelypteris; subgenus: Goniopteris; specificEpithet: rolandii; scientificNameAuthorship: (C.Chr.) R.M.Tryon; **Location:** continent: South America; country: Brazil; countryCode: BR; stateProvince: Bahia; municipality: Ipiaú; verbatimCoordinates: 14°10'50"S, 39°41'33"W; verbatimLatitude: 14°10'50"S; verbatimLongitude: 39°41'33"W; decimalLatitude: -14.180556; decimalLongitude: -39.6925; geodeticDatum: WGS84; **Identification:** identifiedBy: A. Salino; dateIdentified: 2011-05; **Event:** eventDate: 2007-11-13; year: 2007; month: 11; day: 13; **Record Level:** type: specimen; language: Portuguese; collectionCode: BHCB

#### Distribution

**Figure 21.**
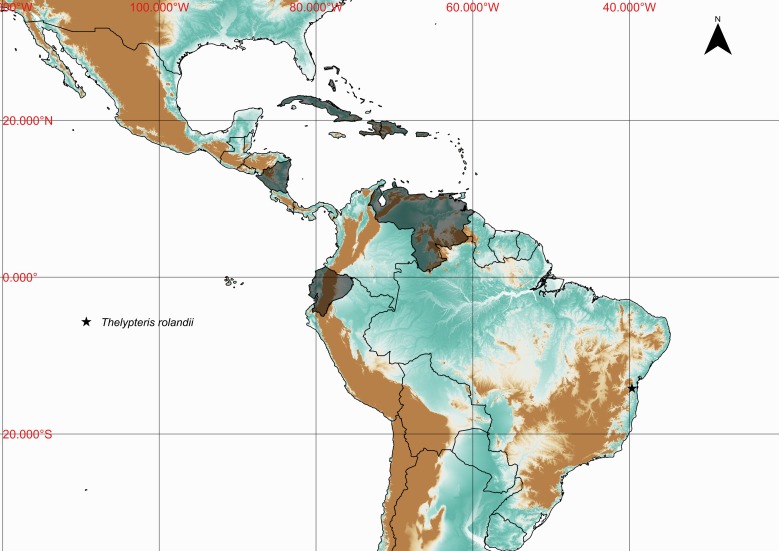
Distribution map of Thelypteris (Goniopteris) rolandii, showing previously known distribution (shaded countries) and new record (star).

Previously known distribution: Antilles, Ecuador, Nicaragua and Venezuela (Smith 1995). Fig. 21.

#### Ecology

Occurs as terrestrial in montane rainforest.

#### Taxon discussion

This species is a putative hybrid between Thelypteris (Goniopteris) tetragona (Sw.) Small and T. (Goniopteris) poiteana (Bory) Proctor (Smith 1983). It can be characterized by the pinnae serrulate to shallowly lobed or incised to 1/3 the distance to costae, 2-3 pairs of basal veins anastomosing below sinus, and the presence of several hairs on sporangial capsule (Smith 1983).

### Thelypteris (Meniscium) arcana

(Maxon & C.V.Morton) C.V.Morton 1967

urn:lsid:ipni.org:names:251217-2

**Figure 22.**
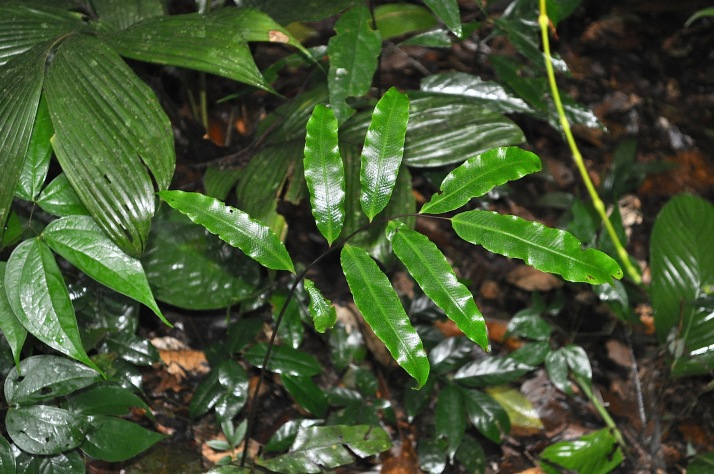
Thelypteris (Meniscium) arcana (Maxon & C.V.Morton) C.V.Morton (Thelypteridaceae).

Thelypteridaceae
Thelypteris (Meniscium) arcana (Maxon & C.V.Morton) C.V.Morton, Contr. U.S. Natl. Herb. 38: 42. 1967. *Dryopteris arcana* Maxon & C.V.Morton, Bull. Torrey Bot. Club 65: 352, t. 11. 1938. Type: Ecuador, *Mexia 7174* (US). Fig. 22.

#### Materials

- **Type status:** Other material. **Occurrence:** catalogNumber: BHCB150026; recordNumber: A. Salino 15026; recordedBy: A. Salino & T.E. Almeida; **Taxon:** taxonID: urn:lsid:ipni.org:names:251217-2; scientificName: Thelypteris (Meniscium) arcana (Maxon & C.V.Morton) C.V.Morton; kingdom: Plantae; class: Polypodiopsida; order: Polypodiales; family: Thelypteridaceae; genus: Thelypteris; subgenus: Meniscium; specificEpithet: arcana; scientificNameAuthorship: (Maxon & C.V.Morton) C.V.Morton; **Location:** continent: South America; country: Brazil; countryCode: BR; stateProvince: Acre; municipality: Mâncio Lima; locality: Parque Nacional da Serra do Divisor, Rio Môa, trail to Cachoeira Formosa; verbatimElevation: 275 m; minimumElevationInMeters: 275; verbatimCoordinates: 07°24'31''S, 73°39'51''W; verbatimLatitude: 07°24'31''S; verbatimLongitude: 73°39'51''W; decimalLatitude: -7.408611; decimalLongitude: -73.664167; geodeticDatum: WGS84; **Identification:** identifiedBy: A. Salino; dateIdentified: 2011-01-10; **Event:** eventDate: 2010-12-13; year: 2010; month: 12; day: 13; **Record Level:** type: specimen; language: Portuguese; collectionCode: BHCB
- **Type status:** Other material. **Occurrence:** catalogNumber: BHCB 150012; recordNumber: A. Salino 15012; recordedBy: A. Salino & T.E. Almeida; **Taxon:** taxonID: urn:lsid:ipni.org:names:251217-2; scientificName: Thelypteris (Meniscium) arcana (Maxon & C.V.Morton) C.V.Morton; kingdom: Plantae; class: Polypodiopsida; order: Polypodiales; family: Thelypteridaceae; genus: Thelypteris; subgenus: Meniscium; specificEpithet: arcana; scientificNameAuthorship: (Maxon & C.V.Morton) C.V.Morton; **Location:** continent: South America; country: Brazil; countryCode: BR; stateProvince: Acre; municipality: Mâncio Lima; locality: Parque Nacional da Serra do Divisor, Rio Môa, trail from Igarapé do Amor; verbatimElevation: 220 m; minimumElevationInMeters: 220; verbatimCoordinates: 07°26'51''S, 73°40'01''W; verbatimLatitude: 07°26'51''S; verbatimLongitude: 73°40'01''W; decimalLatitude: -7.4475; decimalLongitude: -73.666944; geodeticDatum: WGS84; **Identification:** identifiedBy: A. Salino; dateIdentified: 2011-01-10; **Event:** eventDate: 2010-12-13; year: 2010; month: 12; day: 13; **Record Level:** type: specimen; language: Portuguese; collectionCode: BHCB

#### Distribution

**Figure 23.**
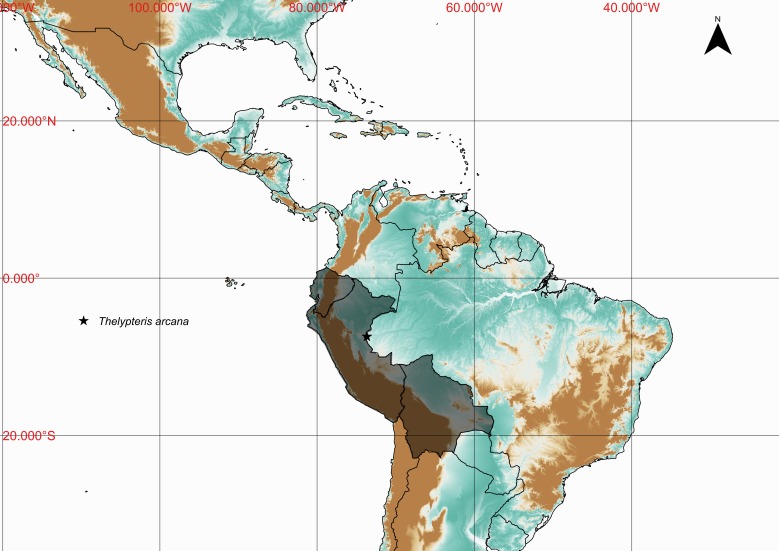
Distribution map of Thelypteris (Meniscium) arcana, showing previously known distribution (shaded countries) and new record (star).

Known distribution: Bolivia, Ecuador and Peru (Tryon et al. 1992). Fig. 23.

#### Ecology

Occurs as terrestrial in lowland rain forest.

#### Taxon discussion

This species is easily recognizable by the 2 – 5 pairs of pinnae, cuneate at base, and tubular yellow to orange glands on the receptacle (Smith 1983). The presence of these glands is shared with T. (Meniscium) andreana (Sodiro) C.V.Morton, the closest species to T. (Meniscium) arcana (Smith 1983).

### Thelypteris (Steiropteris) comosa

(C.V. Morton) C.V. Morton 1961

urn:lsid:ipni.org:names:251274-2

**Figure 24.**
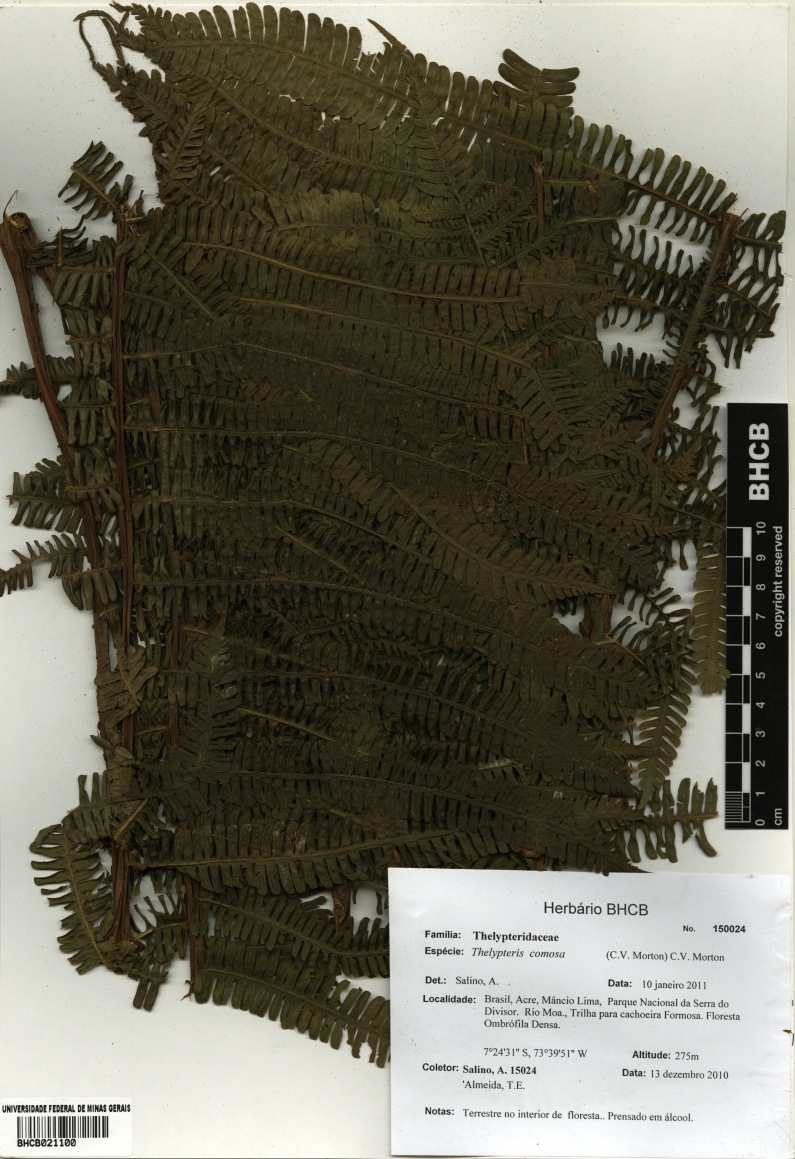
Thelypteris (Steiropteris) comosa (C.V.Morton) C.V.Morton (Thelypteridaceae).

Thelypteris (Steiropteris) comosa (C.V.Morton) C.V.Morton, Amer. Fern. J. 51: 38. 1961. *Dryopteris comosa* C.V.Morton, J. Wash. Acad. Sci. 28: 528. 1983. Type: Peru, *Killip & Smith 25872* (US). Fig. 24.

#### Materials

- **Type status:** Other material. **Occurrence:** catalogNumber: BHCB 150016; recordNumber: A. Salino 15016; recordedBy: A. Salino & T.E. Almeida; **Taxon:** taxonID: urn:lsid:ipni.org:names:251274-2; scientificName: Thelypteris (Steiropteris) comosa (C.V.Morton) C.V.Morton; kingdom: Plantae; class: Polypodiopsida; order: Polypodiales; family: Thelypteridaceae; genus: Thelypteris; subgenus: Steiropteris; specificEpithet: comosa; scientificNameAuthorship: (C.V.Morton) C.V.Morton; **Location:** continent: South America; country: Brazil; countryCode: BR; stateProvince: Acre; municipality: Mâncio Lima; locality: Parque Nacional da Serra do Divisor, Rio Môa, Cachoeira do Ar Condicionado; verbatimElevation: 280 m; minimumElevationInMeters: 280; verbatimCoordinates: 07°27'12"S, 73°41'38"W; verbatimLatitude: 07°27'12"S; verbatimLongitude: 73°41'38"W; decimalLatitude: -7.453333; decimalLongitude: -73.693889; geodeticDatum: WGS84; **Identification:** identifiedBy: A. Salino; dateIdentified: 2011-01-10; **Event:** eventDate: 2010-12-13; year: 2010; month: 12; day: 13; **Record Level:** type: specimen; language: Portuguese; collectionCode: BHCB
- **Type status:** Other material. **Occurrence:** catalogNumber: BHCB 150024; recordNumber: A. Salino 15024; recordedBy: A. Salino & T.E. Almeida; **Taxon:** taxonID: urn:lsid:ipni.org:names:251274-2; scientificName: Thelypteris (Steiropteris) comosa (C.V.Morton) C.V.Morton; kingdom: Plantae; class: Polypodiopsida; order: Polypodiales; family: Thelypteridaceae; genus: Thelypteris; subgenus: Steiropteris; specificEpithet: comosa; scientificNameAuthorship: (C.V.Morton) C.V.Morton; **Location:** continent: South America; country: Brazil; countryCode: BR; stateProvince: Acre; municipality: Mâncio Lima; locality: Parque Nacional da Serra do Divisor, Rio Môa, trail to Cachoeira Formosa; verbatimElevation: 275 m; minimumElevationInMeters: 275; verbatimCoordinates: 07°24'31"S, 73°39'51"W; verbatimLatitude: 07°24'31"S; verbatimLongitude: 73°39'51"W; decimalLatitude: -7.408611; decimalLongitude: -73.664167; geodeticDatum: WGS84; **Identification:** identifiedBy: A. Salino; dateIdentified: 2011-01-10; **Event:** eventDate: 2010-12-13; year: 2010; month: 12; day: 13; **Record Level:** type: specimen; language: Portuguese; collectionCode: BHCB

#### Distribution

**Figure 25.**
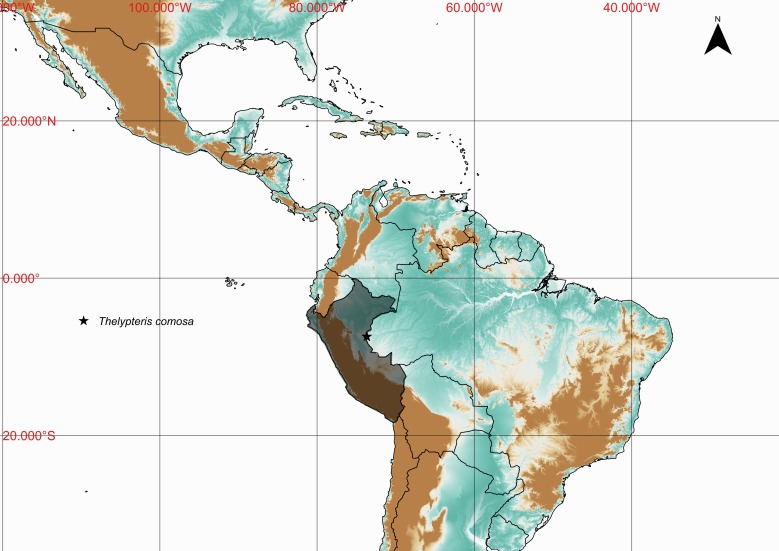
Distribution map of Thelypteris (Steiropteris) comosa, showing previously known distribution (shaded countries) and new record (star).

Previously known distribution: Peru (Tryon et al. 1992). Fig. 25.

#### Ecology

Occurs as terrestrial in montane rain forests.

#### Taxon discussion

This species resembles Thelypteris (Steiropteris) decussata (L.) Proctor, but differs from it by the costae abaxially with dense, soft, septate hairs 1-2 mm long, costules and veins adaxially with dense, strigose hairs up to 2 mm (Tryon et al. 1992).

### Thelypteris (Steiropteris) valdepilosa

(Baker) C.F.Reed 1968

urn:lsid:ipni.org:names:252388-2

**Figure 26.**
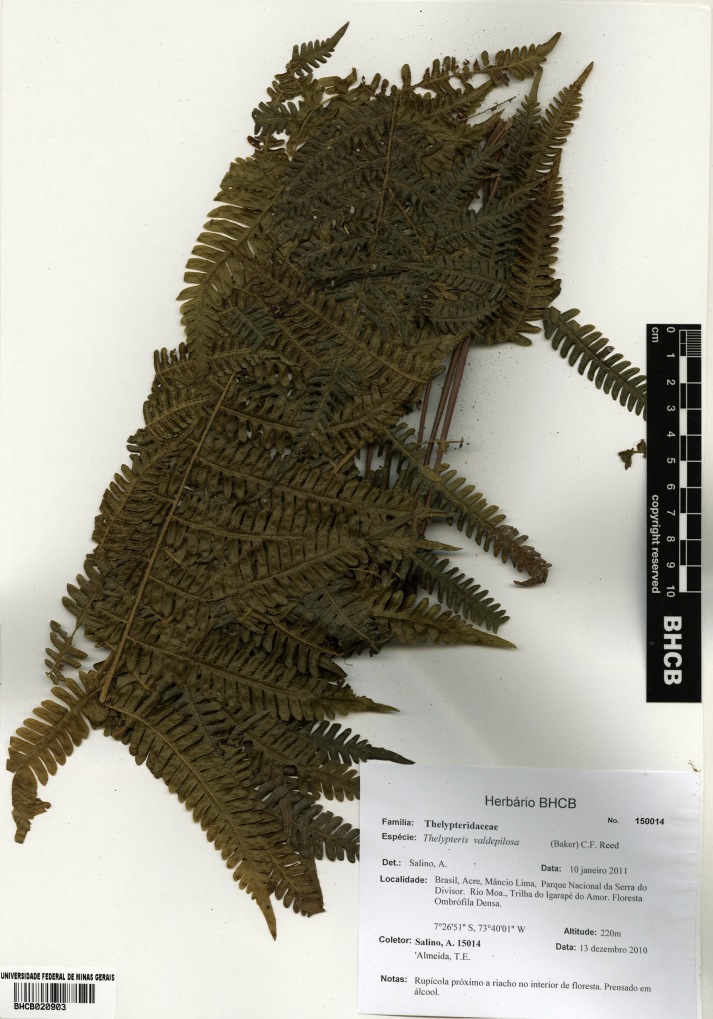
Thelypteris (Steiropteris) valdepilosa (Baker) C.F.Reed (Thelypteridaceae).

Thelypteris (Steiropteris) valdepilosa (Baker) C.F.Reed, Phytologia 17: 323. 1968. *Nephrodium valdepilosum* Baker, J. Bot. 19: 204. 1881. Type: Colombia, *Kalbreyer 1871* (K). Fig. 26.

#### Materials

- **Type status:** Other material. **Occurrence:** catalogNumber: BHCB 150014; recordNumber: A. Salino 15014; recordedBy: A. Salino & T.E. Almeida; **Taxon:** taxonID: urn:lsid:ipni.org:names:252388-2; scientificName: Thelypteris (Steiropteris) valdepilosa (Baker) C.F.Reed; kingdom: Plantae; class: Polypodiopsida; order: Polypodiales; family: Thelypteridaceae; genus: Thelypteris; subgenus: Steiropteris; specificEpithet: valdepilosa; scientificNameAuthorship: (Baker) C.F.Reed; **Location:** continent: South America; country: Brazil; countryCode: BR; stateProvince: Acre; municipality: Mâncio Lima; locality: Parque Nacional da Serra do Divisor, Rio Môa, trail to Igarapé do Amor; verbatimElevation: 220 m; minimumElevationInMeters: 220; verbatimCoordinates: 7°26'51"S, 73°40'01"W; verbatimLatitude: 7°26'51"S; verbatimLongitude: 73°40'01"W; decimalLatitude: -7.4475; decimalLongitude: -73.666944; geodeticDatum: WGS84; **Identification:** identifiedBy: A. Salino; dateIdentified: 2011-01-10; **Event:** eventDate: 2010-12-13; year: 2010; month: 12; day: 13; **Record Level:** type: specimen; language: Portuguese; collectionCode: BHCB

#### Distribution

**Figure 27.**
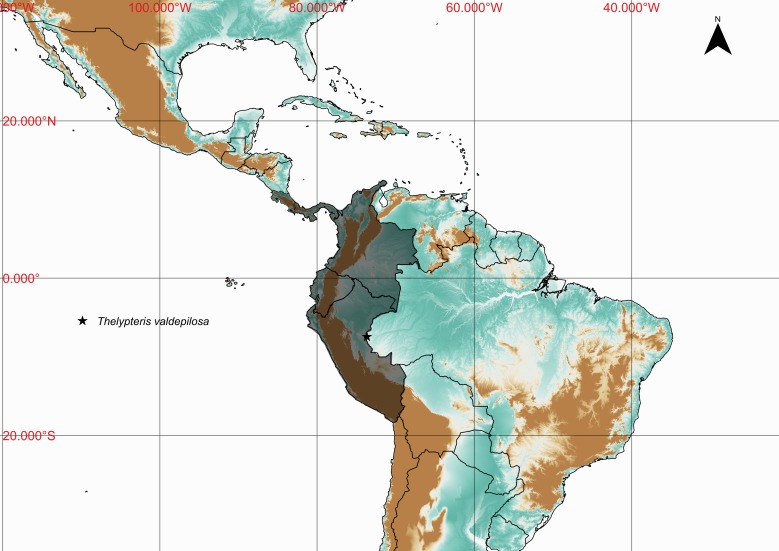
Distribution map of Thelypteris (Steiropteris) valdepilosa, showing previously known distribution (shaded countries) and new record (star).

Previously known distribution: Colombia, Costa Rica, Ecuador, Panama and Peru (Tryon et al. 1992). Fig. 27.

#### Ecology

Occurs as terrestrial in lowland and montane rainforests.

#### Taxon discussion

This species is easily recognizable by the subdimorphic leaves and orangish glands present on receptacle (Smith 1980). The closest species appears to be T. (Steiropteris) leprieurii (Hook.) R.M.Tryon, which can also present dimorphic fronds (Smith 1980).

## Discussion

Species with disjunct ranges occurring in Andes and mountains of eastern Brazil occur in several genera. A few examples of this pattern are *Culcita coniifolia* (Hook.) Maxon, *Jamesonia brasiliensis* Christ, *Eriosorus cheilanthoides* (Sw.) A.F.Tryon (Tryon and Tryon 1982) and *Phlegmariurus aqualupianus* (Spring) B.Øllg. The record of *Alsophila salvinii* is an additional example of floristic relation between the two areas, since this species was previously known to Central America and has been recently recorded in Peru (Smith et al. 2005). Finding new records for Brazilian Atlantic Forest [*Alsophila salvinii*, Thelypteris (Goniopteris) rolandii, *Campyloneurum costatum*] illustrates how much this biodiversity hotspot may still harbors many unknown or poorly known species (Sobral and Stehmann 2009​).

The role of eastern Brazilian mountains in lycophytes and monilophytes diversity and endemism is well known and documented (Tryon 1972, Moran 2008, Salino and Almeida 2008b). Although Moran (1995d), Moran (2008) reports that middle elevations (800 - 2500 m) harbors the most diverse pteridophytes assemblages, in Brazilian Amazon the occurrence of elevations between 200 – 800 m creates an environment completely different from the lowland plains where they are inserted and helps increase species numbers, including endemic and disjunct species. These elevations in Brazilian Amazon, even if low when compared with other mountain regions in Brazil as Serra do Mar, Serra da Mantiqueira or Espinhaço range, stand out from the surrounding matrix to provide environmental, climatic and edaphic conditions to the establishment of a assemblage of species different from the one observed in lowlands (Moran 1995d).

New records presented from Serra do Divisor, Acre, namely *Cyathea subincisa* (Lehnert 2011), *Cyclodium trianae* (Smith 1986), *Elaphoglossum stenophyllum* (Tryon et al. 1991), *Hypoderris brauniana* (Moran et al. 2014), *Pleopeltis stolzei* (Kessler and Smith 2005), *Thelypteris* (M*eniscium*) *arcana*, T. (Steiropteris) comosa, and T. (Steiropteris) valdepilosa (Tryon et al. 1992​), correspond to species that are known to occur nearby in Peru and therefore do not represent disjunct records, but show floristic relations between this mountain range and forests of Amazonian side of the Andes (Daly and Silveira 2008, Obermuller et al. 2014). Some of these species [as Thelypteris (Meniscium) arcana] also occur at low elevations at the Peruvian provinces of Loreto and Pasco (Tryon et al. 1992).

The records found at Pará state are from the mountains of Floresta Nacional de Carajás, where an assemblage of environmental features contributes to the diversity: ferruginous soils at rock outcrops, grasslands and slopes covered with moist forests. These characteristics possibly promote increase of environmental heterogeneity and make possible the establishment of a higher species number. In Amazon region, occurrence of a high species number in a given area appears to be related to the presence of rocky soils (that usually present high values of nutrients) even in areas that do not present mountains but have rough terrain as the Biological Reserve of Uatumã (Zuquim et al. 2008).

## Supplementary Material

XML Treatment for Alsophila salvinii

XML Treatment for Campyloneurum costatum

XML Treatment for Cyathea subincisa

XML Treatment for Cyclodium trianae

XML Treatment for Elaphoglossum stenophyllum

XML Treatment for Hypoderris brauniana

XML Treatment for Pleopeltis stolzei

XML Treatment for Polypodium flagellare

XML Treatment for Tectaria heracleifolia

XML Treatment for Thelypteris (Goniopteris) rolandii

XML Treatment for Thelypteris (Meniscium) arcana

XML Treatment for Thelypteris (Steiropteris) comosa

XML Treatment for Thelypteris (Steiropteris) valdepilosa
